# Histopathological differences between vitiligo and lichen sclerosus et atrophicus using quantitative immunohistochemical analysis

**DOI:** 10.3389/fmed.2023.1205909

**Published:** 2023-07-14

**Authors:** Young Joon Park, Heera Lee, Hyoung Soo Park, You Chan Kim

**Affiliations:** ^1^Department of Dermatology, Ajou University School of Medicine, Suwon, Republic of Korea; ^2^Department of Dermatology, Ewha Womans University College of Medicine, Seoul, Republic of Korea

**Keywords:** lichen sclerosus, vitiligo, melanin, pigmentation, melanocytes

## Abstract

**Introduction:**

Lichen sclerosus et atrophicus (LS) is rare skin condition characterized by the presence of whitish patches primarily affecting the genital and perianal areas, though it can occur other parts of the body. LS may result in skin depigmentation without textural changes and should be differentiated from vitiligo. However, the histopathological features of hypopigmentation during vitiligo and LS have rarely been compared and have not been precisely described using quantitative immunohistochemical analysis. This study, therefore, aimed to investigate and compare the pigmentary characteristics of LS and vitiligo lesions using histochemical and immunohistochemical staining.

**Methods:**

We included 31 and 46 patients diagnosed with LS and vitiligo, respectively, at Ajou University Hospital between March 2009 and March 2020 in this study. Their medical charts and skin biopsy specimens were retrospectively reviewed. Additionally, Fontana–Masson staining for melanin and immunohistochemical staining for Melan-A, NKI/beteb, tyrosinase, and microphthalmia-associated transcription factor was performed.

**Results:**

The melanin content, as well as the number of melanocytes was, in general, significantly higher in the epidermis of patients in the LS group compared with that in the vitiligo group. However, 22.6% of LS tissues showed less melanin pigmentation, 25.8% of LS specimens exhibited a lower number of melanocytes, and 29.0% of LS specimens demonstrated less melanocyte activity when compared with the average of vitiligo specimens.

**Conclusion:**

As lower melanin pigmentation and the near absence number of melanocytes were also observed in several LS specimens, both the clinical and histological findings must be comprehensively reviewed to differentiate vitiligo from LS.

## Introduction

Lichen sclerosus et atrophicus (LS), first reported in 1887, is a chronic inflammatory dermatosis that commonly involves mucocutaneous lesions commonly observed in the anogenital and extragenital areas (1). Despite its typical clinical features are porcelain-like white patches or plaques with atrophic lesions, fissures, erythema, and sclerosis (Supplementary Figure S1A) (2), LS may also show significant depigmentation without textural changes, thereby mimicking vitiligo (Supplementary Figure S1B). As the treatment and prognosis of these two diseases differ significantly, it is crucial to accurately distinguish LS from vitiligo (3).

The mechanism of vitiligo involves an autoimmune attack against melanocytic antigens and subsequent destruction of melanocytes (4). Subsequent complete loss of melanocytes in histopathology is a typical characteristic of vitiligo (3). Recent studies have suggested that autoimmune mechanisms may play a major role in LS development, similar to vitiligo (5). The mechanism underlying depigmentation of LS is believed to be associated with either the blockage of melanin transfer to keratinocytes or the destruction of melanocytes during lichenoid inflammation (6). However, the histopathological features of hypopigmentation during vitiligo and LS have rarely been compared and have not been precisely described using quantitative immunohistochemical analysis. Therefore, we investigated and compared the histological characteristics of LS and vitiligo lesions.

## Materials and methods

### Patients

We retrospectively evaluated electronic medical records and skin biopsy specimens of 31 and 46 patients diagnosed with LS and vitiligo, respectively, who visited our hospital between March 2009 and March 2020. Patients who were clinically diagnosed with either LS or vitiligo and who had undegone skin biopsy for confirmation were included in the study. At the first visit, Wood’s light examination was performed on all enrolled patients presenting with a hypomelanotic patch or plaque on the genital or extragenital area. A vitiligo diagnosis was confirmed based on the following criteria: (1) presence of well-defined hypopigmented patches with or without poliosis, (2) marked accentuation under Wood’s light, (3) absence of melanocytes observed in histopathological evaluation, and (4) positive response to treatment with excimer laser and/or narrow band UVB therapy. On the other hand, patients were confirmed as LS based on the following: (1) whitish glossy patches, (2) current or past history of itching, (3) evident epidermal spongiosis and homogenized collagen fibers present in histopathology, and (4) favorable response to topical or intralesional steroids. Based on these criteria, dermatologists finalized the diagnosis. Informed consent was obtained from all the patients for the use of clinical photographs and biopsy samples. This study was approved by the Institutional Review Board of Ajou University Hospital (IRB-No: AJIRB-MED-EXP-18-404). Clinical data, such as age, sex, and location of skin lesions, were collected by reviewing the electronic medical records.

### Histopathological analysis

All skin biopsy specimens were independently examined by a dermatopathologist (YCK) and a dermatologist (YJP) using conventional hematoxylin and eosin (H&E) staining for general histopathological changes and Fontana–Masson staining for melanin deposition. Immunohistochemical staining was performed using Melan-A (1,100 dilution, Thermo Scientific, Fremont, CA, United States), NKI/beteb (1,10 dilution, Monosan, Uden, Netherlands), microphthalmia-associated transcription factor (MITF) (1,10 dilution, Cell Marque, Rocklin, CA, United States), and tyrosinase (1,200 dilution, Thermo Scientific). Color red was used to indicate positivity. Image analysis was performed using Image Pro Plus Version 4.5 (Media Cybernetics Co., Silver Spring, Rockville, MD, United States). The amount of melanin pigment was evaluated as the ratio of pigmented area (PA) to measured epidermal area (EA). Melan-A, NKI/beteb, and tyrosinase expression was calculated as the ratio of the stained area to the measured EA (SA/EA). The number of melanocytes that were positively stained for MITF per 1 mm of basal epidermis (MC/1B) was counted manually. The length of the basal epidermis was estimated using image analysis. Each melanocyte was counted as one cell when its nucleus was confirmed using MITF immunohistochemical staining. Each measurement was evaluated under constant magnification (×200).

### Statistical analysis

SPSS Statistics version 20 (IBM, Armonk, NY, United States) was used for the statistical analyses. The association between the presence of abnormal skin tissue findings on H&E staining and disease was verified using chi-square test. Unpaired *t*-test was used to verify the statistical significance between the immunohistochemical staining of the two groups. Statistical significance was set at *p* < 0.05.

## Results

### Clinical features

Of the 77 patients, 31 (26 women, 83.9%) had LS and 46 (33 women, 71.7%) had vitiligo. The median age of patients with LS was 49.2 (range: 4–80) and that of patients with vitiligo was 41.5 (range: 0–76). In the LS group, hypopigmentation was observed on the face in one patient (3.2%), on the trunk in two patients (6.5%), on the extremities in one patient (3.2%), and in the genital area in 27 patients (87.1%). In the vitiligo group, 19 patients (41.3%) had lesions on the head and neck, 16 (34.8%) had lesions on the extremities, 10 (21.7%) had lesions on the trunk, and one patient (2.2%) had lesions on the genitalia. The duration of disease varied from decades to months, with a median duration of 3.2 years and 1 year for LS and vitiligo, respectively. Fifteen patients (48.4%) with LS complained of pruritus, and none of the vitiligo patients had an itching sensation. The clinical characteristics of the patients are summarized in Table 1.

**Table 1:** Demographics and clinical characteristics of the patients.

Characteristics	LS	Vitiligo
No. of cases	31	46
Sex, *n* (%)		
Male	5 (16.1%)	13 (28.3%)
Female	26 (83.9%)	33 (71.7%)
Age, median (range), y	49.2 (4–80)	41.5 (0–76)
Site of lesion, *n* (%)		
Face and neck	1 (3.2%)	19 (41.3%)
Trunk	2 (6.5%)	10 (21.7%)
Extremities	1 (3.2%)	16 (34.8%)
Genital area	27 (87.1%)	1 (2.2%)
Disease duration, median (range), y	3.2 (0.5–30)	1 (0.3–8)
Associated symptom, *n* (%)		
Pruritus	15 (48.4%)	0
Wood’s light positive results, *n* (%)	7 (22.5%)	36 (82.6%)

### Histopathological characteristics

**Figure 1:**
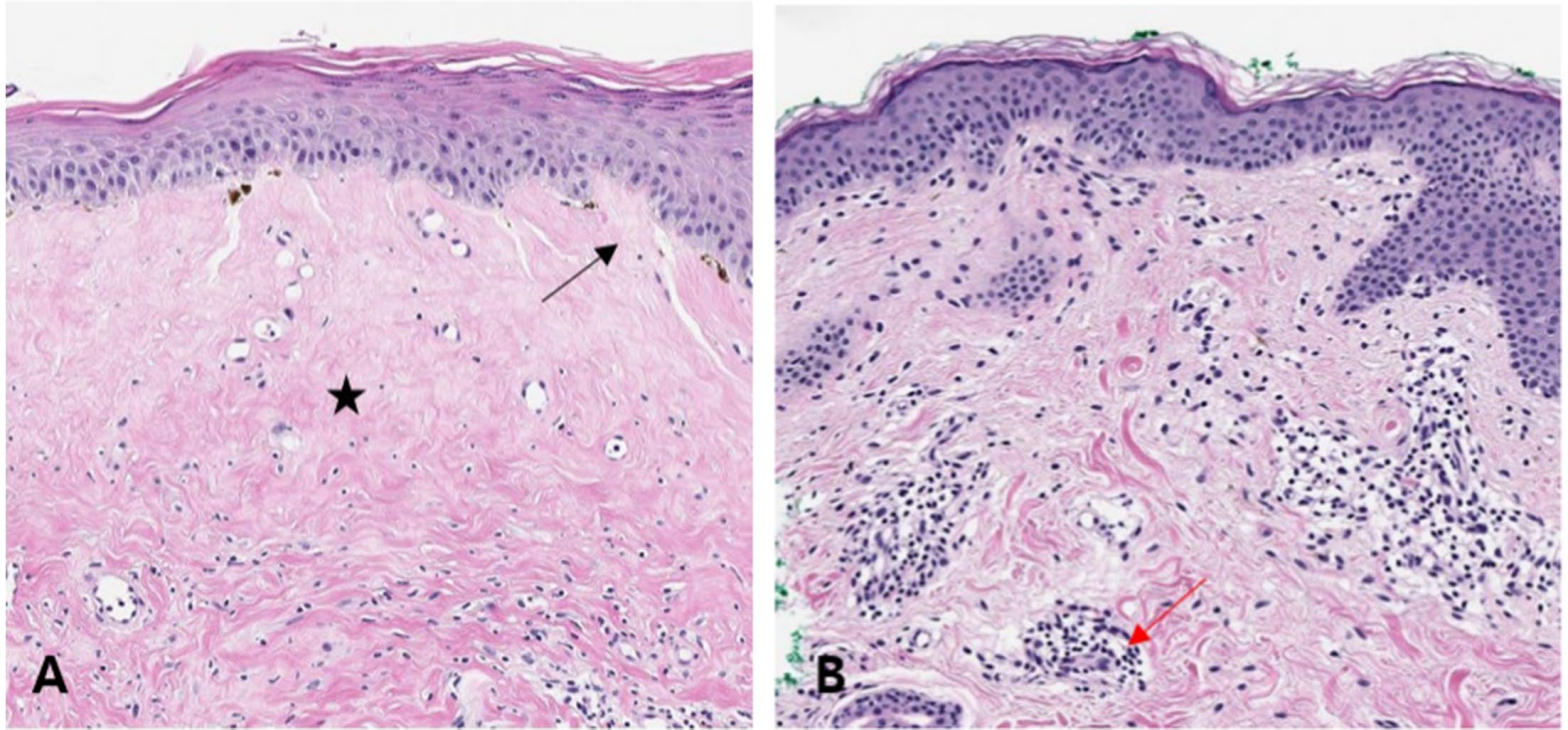
Hematoxylin–eosin (H&E) stained tissues of lichen sclerosus (LS) and vitiligo Histopathological features of LS and vitiligo are shown. This demonstrates epidermal spongiosis (black arrow) with homogenized collagen fibers (black star) in the upper dermis, suggesting LS [**(A)**, H&E, ×200]. Histopathological features of mild perivascular inflammation (red arrow), along with reduced pigmentation in a vitiligo sample [**(B)**, H&E, ×200].

H&E staining revealed significant epidermal alterations and lichenoid or diffuse inflammation in the LS cases (Table 2). Epidermal atrophy, epidermal spongiosis, lichenoid lymphocyte infiltration, and homogenized collagen fibers were observed in the LS group but not in the vitiligo group (Figure 1A). Perifollicular inflammation was observed more frequently in patients with vitiligo than in those with LS (Figure 1B).

**Table 2:** Histopathological characteristics of lichen sclerosus (LS) and vitiligo (hematoxylin & eosin stain).

Characteristics	LS	Vitiligo	*p*-value
Epidermal atrophy	23 (74.2%)	2 (4.3%)	0.000
Epidermal spongiosis	15 (48.4%)	0	0.000
Lichenoid inflammatory cell infiltration	21 (67.7%)	1 (2.1%)	0.000
Dilated vessels	19 (61.3%)	5 (10.9%)	0.000
Perivascular inflammation	14 (45.2%)	23 (50%)	0.682
Perifollicular inflammation	5 (16.1%)	22 (47.8%)	0.004
Homogenized collagen fiber	21 (67.7%)	0	0.000

Skin tissue findings on H&E staining and disease was verified using chi-square test. Statistical significance was set at *p* < 0.05.

### Quantitative comparison of epidermal and dermal melanin content in LS and vitiligo lesions

**Figure 2:**
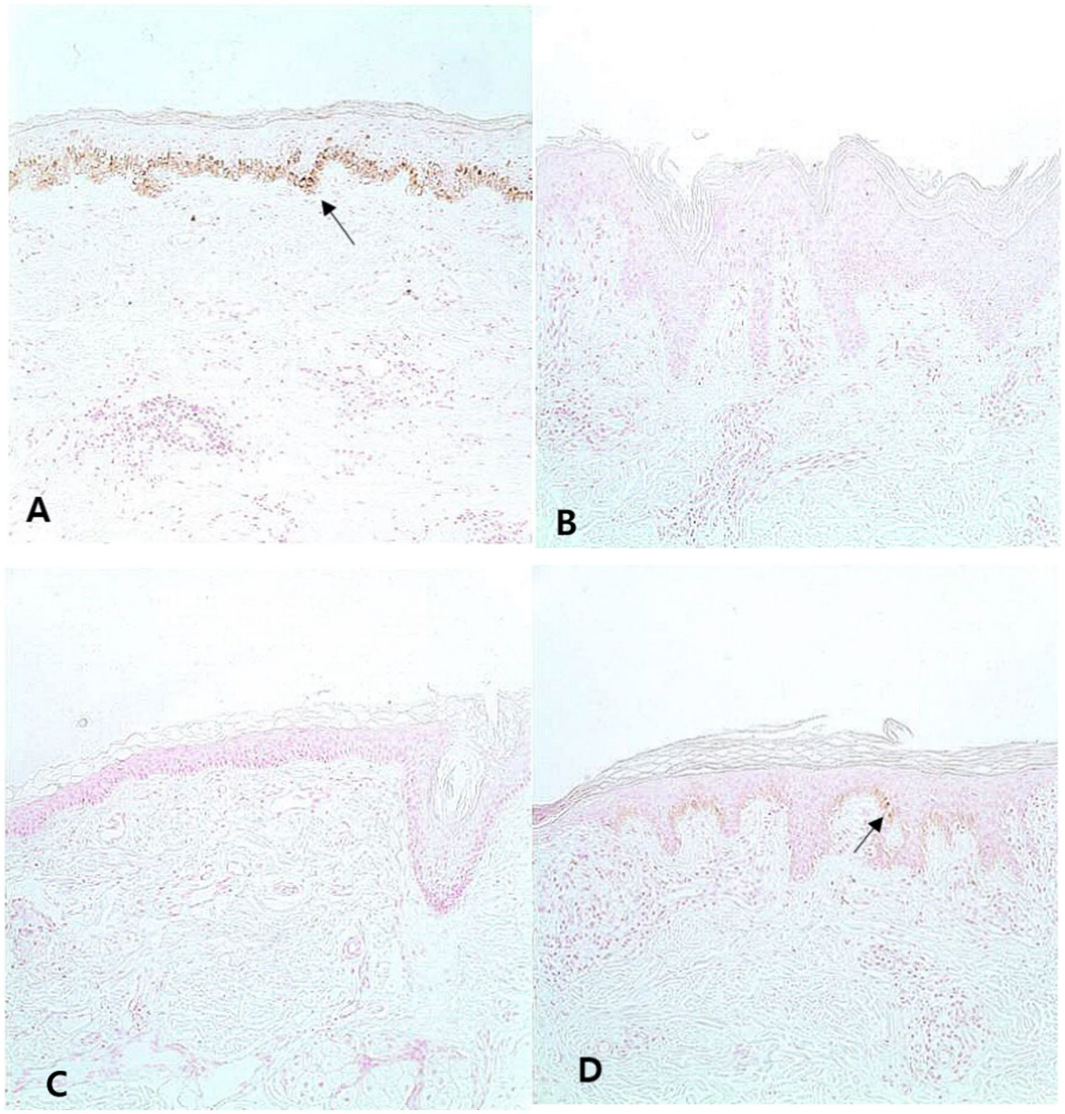
Melanin pigment in the lesion of lichen sclerosus (LS) and vitiligo Typical LS samples show melanin in the basal layer [**(A)**, Fontana–Masson, ×200]. In addition, most vitiligo specimens do not contain melanin [**(B)**, Fontana–Masson, ×200]. However, some LS samples contained fewer melanin than the average melanin of vitiligo samples [**(C)**, Fontana–Masson, ×200]. Some vitiligo samples show more melanin than LS samples [**(D)**, Fontana–Masson, ×200]. Arrows indicate melanin.

Fontana–Masson (FM) staining revealed that, while both showed decreased melanin pigment in the epidermis, the pigmentation of vitiligo patients was significantly lower than that of LS patients (Figures 2A,B, [PA/EA] 0.024 ± 0.004 vs. 0.065 ± 0.013, *p* = 0.008). However, 22.6% of LS patients exhibited less melanin pigmentation (Figure 2C) than the average amount in vitiligo patients (Table 3), whereas a few vitiligo samples showed more melanin than the average amount in LS patients (Figure 2D), indicating that the degree of pigmentation should not be the sole factor in differentiating LS from vitiligo.

**Table 3:** Lichen sclerosus (LS) patients showing less melanin or less melanocyte activity than the average amount of melanin or melanocyte activity in patients with vitiligo.

Stain	*n*	%
Fontana-Masson	7/31	22.6
MITF	8/31	25.8
NKI/beteb	17/31	54.8
Melan-A	9/31	29.0
Tyrosinase	2/31	6.5

MITF, microphthalmia-associated transcription factor.

### Quantitative comparison of the number of epidermal melanocytes and melanogenic activity in LS and vitiligo

**Figure 3:**
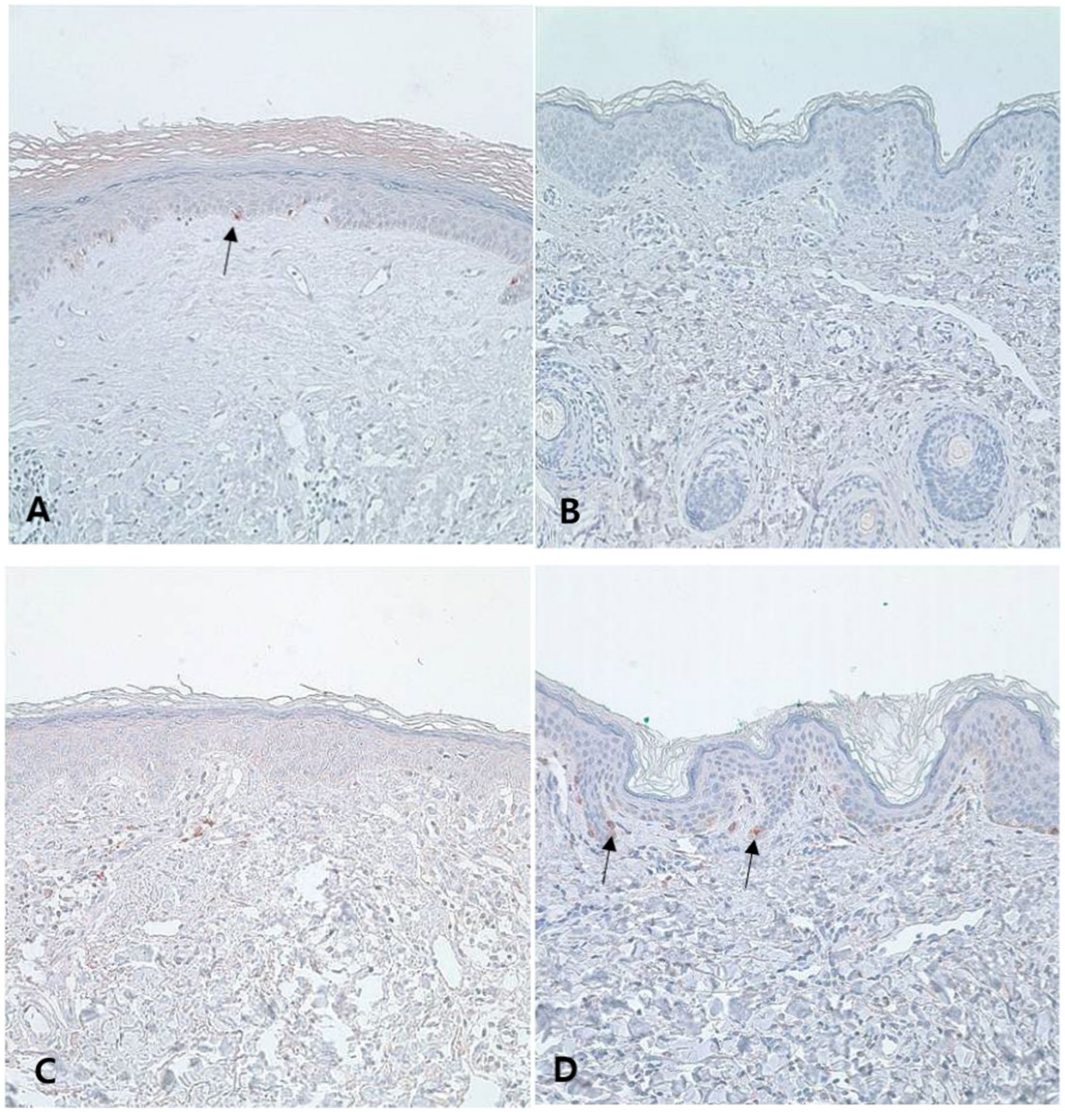
Number of melanocytes in the lesion of lichen sclerosus (LS) and vitiligo Typical LS samples show some melanocytes in the basal layer [**(A)**, Melan-A, ×200], and most of the vitiligo samples have sparse melanocytes [**(B)**, Melan-A, ×200]. However, some LS samples have fewer melanocytes than the average amount of vitiligo [**(C)**, Melan-A, ×200], and some vitiligo samples show more melanocytes than LS samples [**(D)**, Melan-A, ×200]. Arrows indicate melanocyte.

Melan-A staining was used to count the number of melanocytes (Figures 3A,B). LS lesions showed a significantly higher number of Melan-A-positive melanocytes than vitiligo lesions (SA/EA: 0.007 ± 0.007 vs. 0.003 ± 0.003, *p* < 0.0001). MITF staining also showed significantly more melanocytes in LS than in vitiligo (MC/1B: 0.008 ± 0.006 vs. 0.004 ± 0.005, *p* < 0.0001). Similar to the FM staining results, 29.0% of the LS samples exhibited a lower number of melanocytes than the average number of melanocytes observed in vitiligo samples (Figures 3C,D; Table 3).

**Figure 4:**
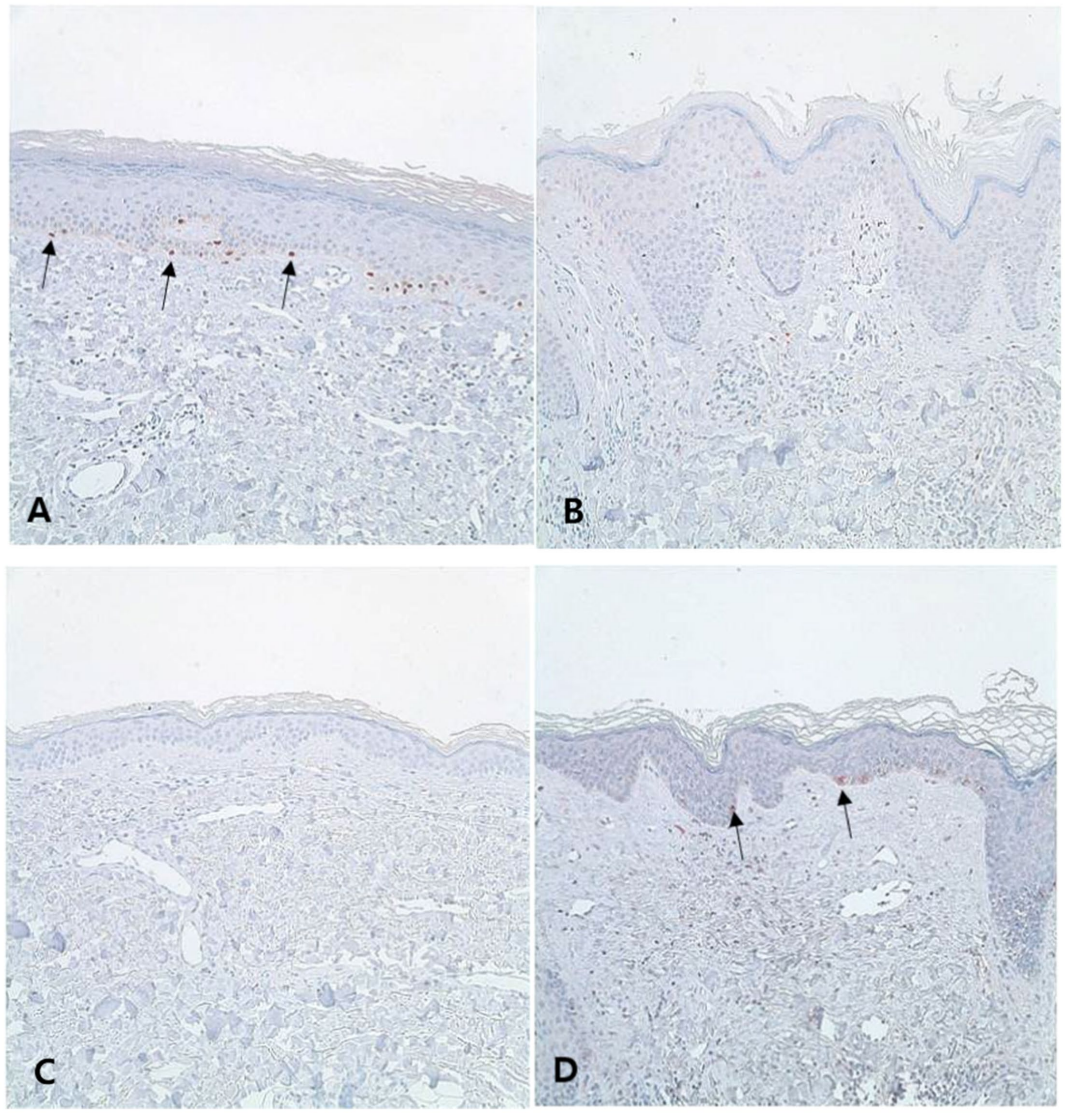
Melanocytic activities of lichen sclerosus (LS) and vitiligo Typical LS samples have some melanocyte activity in the basal layer [**(A)**, tyrosinase, ×200], whereas vitiligo samples scarcely show melanocyte activity [**(B)**, tyrosinase, ×200]. However, some LS samples exhibit less melanocyte activity than the average melanocyte activity observed in vitiligo samples [**(C)**, tyrosinase, ×200], and some of the vitiligo samples show more melanocyte activity than the LS samples [**(D)**, tyrosinase, ×200]. Arrows indicate melanocyte.

Tyrosinase (a marker for melanogenic activity)-positive melanocytes were also significantly more frequently detected in most LS lesions than in vitiligo lesions (Figures 4A,B; SA/EA: 0.007 ± 0.005 vs. 0.003 ± 0.004, *p* < 0.0001). Only 6.5% of the LS samples showed less tyrosinase staining than the average of the vitiligo samples (Figures 4C,D).

NKI/beteb expression, another melanocyte lineage-specific marker (SA/EA: 0.004 ± 0.003 vs. 0.003 ± 0.004, *p* = 0.246), was not significantly different between the two groups (Table 3). Moreover, more than half of the LS samples showed less NKI/beteb staining than the average amount in the vitiligo samples (Table 3).

## Discussion

In this study, we differentiated the two conditions based on (1) clinical findings, (2) Woods’ light accentuation, (3) histopathologic findings, and (4) response to the treatment. Although (1) and (2) are typically available in a general clinical setting, our results demonstrate that these alone might not be sufficient for more challenging cases.

Table 1 presents the multiple clinical characteristics that can aid in differentiating between the two diseases. For instance, while LS primarily involves the anogenital area, vitiligo does not exhibit such a preference. The texture of LS and vitiligo lesions can also differ, with LS typically presenting porcelain-like sclerotic patches, while vitiligo usually does not cause any textural change. Wood’s light examination may also help in differential diagnosis, as most vitiligo patients (82.6%) presented with marked accentuation in our study. Otherwise, most patients with LS (77.5%) showed only mild accentuation under Wood’s light. Other than Wood’s light, dermoscopy findings might help differentiating LS from vitiligo. Specific features of LS show refractile white strands due to collagen in the dermis with prominent vascular structure (7). Vitiligo sample show white glow due to the absence of pigment network (8). Unfortunately, we did not possess a detailed data on dermoscopy findings of our patients for appropriate analysis. However, performing a detailed clinical examination including dermoscopy and Wood’s light test on the anogenital region may be difficult in some clinical settings. Also, vitiligo lesions on genitalia may not show marked accentuation under Wood’s light. In our study, we confirmed the diagnosis of LS and vitiligo by response to therapy, but it was only possible because our study was a retrospective study. Thus, skin biopsy remains a major option for those who need to differentiate between the two diseases.

In H&E staining, only LS samples showed a significantly high rate of atrophy and spongiosis of the epidermis, homogenized collagen fibers in the dermis, and lichenoid cell infiltration. However, not all LS samples possess such characteristic histological traits. Further, as melanocyte destruction and depigmentation are key histologic findings in vitiligo, we also performed IHC. Many previous studies have concluded that long-standing vitiligo patches usually show an absence of melanin and a definite loss of melanocytes in the epidermis (9). Other studies have reported the presence of melanocytes and melanin pigments in vitiligo for a long (up to 25 years) duration (4, 10). In our study, most vitiligo samples showed less epidermal melanin components and less melanocyte activity than the LS tissue samples; however, some vitiligo specimens contained active melanocytes with melanin pigments. In detail, 22.6% of LS samples exhibited less melanin pigments following Fontana–Masson staining, and 54.8% of LS samples of NKI/beteb staining showed less melanocyte activity than the average values observed in vitiligo samples. Interestingly, tyrosinase staining showed the most significant difference in melanocytes between LS and vitiligo (Tables 3, 4). We have previously reported that it is occasionally difficult to use typical histopathological features when distinguishing LS from vitiligo (11). Based on our results, we recommend the use of tyrosinase as an appropriate immunohistochemical marker to differentiate between the two diseases.

**Table 4:** Quantitative comparison of the melanocyte-related markers between lichen sclerosus (LS) and vitiligo.

Stain	LS	Vitiligo	*p*-value
MITF (MC/1B)	0.008 ± 0.006 (0 ~ 0.028)	0.004 ± 0.005 (0 ~ 0.005)	< 0.0001
Melan-A (SA/EA)	0.007 ± 0.007 (0 ~ 0.026)	0.003 ± 0.003 (0 ~ 0.012)	< 0.0001
NKI/beteb (SA/EA)	0.004 ± 0.003 (0 ~ 0.015)	0.003 ± 0.004 (0 ~ 0.010)	0.246
Tyrosinase (SA/EA)	0.007 ± 0.005 (0 ~ 0.023)	0.003 ± 0.004 (0 ~ 0.009)	< 0.0001

The independent *t*-test was used to verify the statistical significance. Statistical significance was set at *p* < 0.05. Mean value with standard deviation and range have been shown. MITF, microphthalmia-associated transcription factor; MC/1B, number of melanocytes stained for MITF per 1 mm length of basal epidermis; SA/EA, stained area per epidermal area.

Vitiligo is known to be an autoimmune disease (4). There is no consensus on the mechanisms that underlie LS, but there have been suggestions that LS is mainly caused by an autoimmune reaction (6). Antibodies against melanocytes as well as cytotoxic T cells play a role in the pathogenesis of vitiligo (12). Also, antibodies against extracellular matrix protein 1 and basement membrane zone components, especially BP 180, are thought to be the cause of LS. In addition, some cases involving co-localization of LS and vitiligo reported recently could suggest the relation between two diseases (13, 14). Another case report of simultaneous LS and vitiligo suggested lichenoid dermatitis as a factor triggering an autoimmune reaction to melanocytes (15). Thus, dermatologists should always be aware of the possibility of these two diseases occurring together.

A major limitation of our study is that most of the tissue samples of patients with LS were obtained from the genital area, whereas diverse areas of skin were used in vitiligo patients. As the degree of pigmentation, as well as other histologic characteristics, differ according to the site of the skin, it should be noted that our study only provides a general histologic difference between the two diseases. Histopathologic findings including IHC, undoubtedly aid in the differentiating between the two conditions. However, dermatologists should not rely solely on any one aspect such as IHC. Instead, they should comprehensively consider all clinical and histopathological findings, especially in challenging cases.

## Data availability statement

The original contributions presented in the study are included in the article/Supplementary material, further inquiries can be directed to the corresponding author.

## Ethics statement

The studies involving human participants were reviewed and approved by Institutional Review Board of Ajou University Hospital (IRB-No: AJIRB-MED-EXP-18-404). Written informed consent for participation was not required for this study in accordance with the national legislation and the institutional requirements.

## Author contributions

YP, HL, HP, and YK contributed to the study conception and design, material preparation, data collection, analysis, and commented on previous versions of the manuscript. The first draft of the manuscript was written by YP and HL. All authors contributed to the article and approved the submitted version.

## Funding

This work was supported by the Basic Science Research Program through the National Research Foundation of Korea (NRF) funded by the Ministry of Education (grant number NRF-2022R1I1A1A01069145).

## Conflict of interest

The authors declare that the research was conducted in the absence of any commercial or financial relationships that could be construed as a potential conflict of interest.

## Publisher’s note

All claims expressed in this article are solely those of the authors and do not necessarily represent those of their affiliated organizations, or those of the publisher, the editors and the reviewers. Any product that may be evaluated in this article, or claim that may be made by its manufacturer, is not guaranteed or endorsed by the publisher.
