# Supplementary material for: Histopathological differences between vitiligo and lichen sclerosus et atrophicus using quantitative immunohistochemical analysis
Source: Front Med (Lausanne). 2023 Jul 14;10:1205909. doi: 10.3389/fmed.2023.1205909 (PMC10376688; doi:10.3389/fmed.2023.1205909)
Supplement: Supplementary file 1 [file Data_Sheet_1.docx]

**Supplementary Figure Legend**

Supplementary Figure 1. Clinical photographs of lichen sclerosus (A) Lesion showing glossy and whitish patches with mild atrophy. (B) Lesion displaying less texture change, and rather presenting erythema.


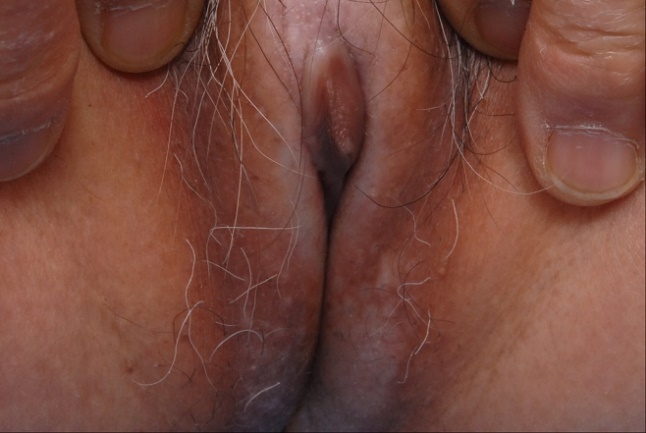

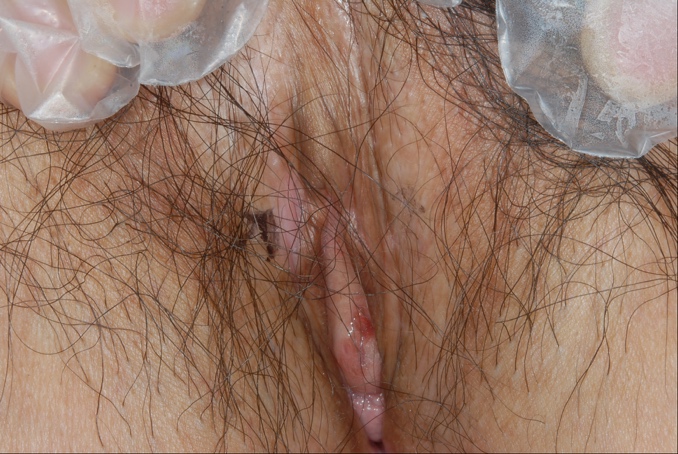


**B**

**A**
